# Perceptions of gender-based discrimination during surgical training and practice

**DOI:** 10.3402/meo.v20.25923

**Published:** 2015-02-03

**Authors:** Adrienne N. Bruce, Alexis Battista, Michael W. Plankey, Lynt B. Johnson, M. Blair Marshall

**Affiliations:** 1 School of Medicine, Georgetown University Medical Center, Washington, DC, USA; 2 MedStar Health Research Institute, Hyattsville, MD, USA; 3 Division of General Surgery, Department of Surgery, MedStar Georgetown University Medical Center, Washington, DC, USA; 4 Division of Thoracic Surgery, Department of Surgery, MedStar Georgetown University Medical Center, Washington, DC, USA

**Keywords:** gender discrimination, women, sexual harassment, surgery, work discrimination, women in medicine

## Abstract

**Background:**

Women represent 15% of practicing general surgeons. Gender-based discrimination has been implicated as discouraging women from surgery. We sought to determine women's perceptions of gender-based discrimination in the surgical training and working environment.

**Methods:**

Following IRB approval, we fielded a pilot survey measuring perceptions and impact of gender-based discrimination in medical school, residency training, and surgical practice. It was sent electronically to 1,065 individual members of the Association of Women Surgeons.

**Results:**

We received 334 responses from medical students, residents, and practicing physicians with a response rate of 31%. Eighty-seven percent experienced gender-based discrimination in medical school, 88% in residency, and 91% in practice. Perceived sources of gender-based discrimination included superiors, physician peers, clinical support staff, and patients, with 40% emanating from women and 60% from men.

**Conclusions:**

The majority of responses indicated perceived gender-based discrimination during medical school, residency, and practice. Gender-based discrimination comes from both sexes and has a significant impact on women surgeons.

The most recent Association of American Medical Colleges Physician Specialty Data Book, based on 2010 census statistics, reports that women currently represent, on average, 50% of annual graduating medical students, 46% of residents/fellows, but only 30% of all active physicians (1). Despite the relatively similar numbers of women and men graduating from medical school, women continue to be particularly underrepresented in surgery and the surgical subspecialties. Women comprise only 36 and 15% of residents and active physicians, respectively, in general surgery, compared to 45 and 34% in internal medicine (1). Although numbers of women in medical school and surgical residencies are increasing, the low number of women surgeons in practice continues to be of concern, as women consistently make up one of the lowest percentages of the surgical profession as compared to other specialties (1). Studies identifying factors related to this gap are limited.

Longer training hours and issues relating to work, family, and lifestyle have been touted by some experts as the primary causes keeping women from entering surgical fields. Female and male medical students are consistently found to have equal interest in general surgery, but female students often perceive a role strain between family and professional life (2). Yet, this perception is not limited to female medical students. Men are just as likely as women to report that quality of lifestyle dissuades them from choosing surgery; in fact, surgical workload and family concerns appear to be less of a deterrent for women (3). Despite these noted perceptions of medical students, female surgeons report a significantly different and more positive view of their career choice, with the majority of surgeons reporting satisfaction at comparable rates to other specialties (2). Women within the surgical field report a satisfactory degree of control over their lifestyle despite what is perceived by female medical students (3). Rather, the belief that lifestyle factors deter female medical students from a career in surgery is more rooted in a discordance between perception and reality of the field, suggesting a component of miscommunication within the education structure (2). This begs the question: What factors do keep women from pursuing a surgical career?

Previous literature reports that women are more likely than men to avoid the field of surgery based on perceptions of surgical personalities and persistence of the ‘old boys’ club’ mentality (4–6). Although the belief that gender discrimination exists may be outdated in modern society, medical students continue to report the most gender-based discrimination on surgery rotations, a trend that has had similar incidence across the last decade (6–12). Because this window into surgical life is medical students’ primary exposure to a future in surgery, students and health care professionals alike are right to consider whether gender-based discrimination is a deterrent to pursuit of a surgical career. Still, the argument can be made that medical students’ perceptions of mistreatment is a spectrum with many falling outside of what is considered official mistreatment by institutional policies (13). However, it is important to note that overall perception of mistreatment, even for the educational purposes, is negative in terms of the well-being of students and the profession; empowered students are more involved, motivated, and committed to their teachers and patients (13). As hospitals trend toward a gradual drift away from their respective medical schools, newer clerkships in departments of surgery may not always fall under academic guidelines nor have a vested interest in medical education. Whether this hampers progress toward fairness remains to be seen.

Female surgeons further along in their careers are leaving their professions altogether, despite reports of high self-efficacy (confidence in leadership and surgical skills) at career onset. Reported reasons for attrition include stymied career advancement, low salaries, and chair/leadership issues (7). In surgery, compared to other fields of medicine, women receive less personal support and career advancement, as well as markedly lower salaries, despite practice settings, work hours, and years out of training (8, 14). In fact, the percent of female leaders in academic surgery remains the lowest of all of the academic medicine departments (15). Because of perceived barriers, female resident and physician attrition rates remain significantly higher than male colleagues’ rate both in surgery and within other specialties, including internal medicine, obstetrics and gynecology, and anesthesiology (16). Of note, no sex differences are observed in degree of confidence or dissatisfaction of career (7). Despite women in academic surgery feeling especially well-prepared and competent to excel in their fields when starting out, they later perceive their most significant career barrier as ‘interactional bias’, defined as subtle patterns of discriminatory exchanges (7). Most notably, female faculty winds up with more negative perceptions than male faculty about gender-based hindrances to career progress (9).

Medical schools, surgical societies, and other institutions have attempted to improve this situation through programs to support women in surgery and academia. It remains to be seen, however, whether discrimination in the surgical workplace, whether perceived or experienced, overt or covert, has diminished. In order to better understand perceived discrimination in today's surgical workplace, we surveyed women in the national Association of Women Surgeons (AWS). For the purpose of our study, we defined overt discrimination to include harmful or intimidating actions or utterances directed at the subject (e.g., sexual advances or inappropriate comments specifically related to gender). Covert discrimination, by comparison, was defined to include subtle inequalities or treatments directed at hindering the subject professionally (e.g., double standards, discouragement, or biased referral patterns). We designed our survey to determine the prevalence of gender-based discrimination in a variety of hospital environments and to ascertain offenders (source) and targets (recipients) of discrimination in each. We also distinguished between the more traditional male–female brand of discrimination (inter-sex) and discrimination that result when women disparage or discourage other women (same-sex).

Most research on discrimination in surgery has focused on academic medicine and plastic surgery and has investigated how women's perceptions compare to those of men (6–8). Little is known of prevalence rates at various stages of a surgical career (i.e., medical education vs. residency vs. practice), relative prevalence of inter-sex vs. same-sex discrimination, specific sources of discrimination in the surgical environment, and frequency of occurrence, nor the implications of these perceived discriminatory actions on surgical career. In our study, we attempt to broadly categorize the scope and nature of perceived gender-based discrimination, hypothesizing that gender-based discrimination exists at all stages and settings of a surgical career and that both inter-sex and same-sex discrimination factor in discouraging women from entering surgical fields.

## Methods

### Population

We developed a survey to address perceptions of discrimination during medical school, surgical training, and practice. The survey was piloted with female general surgery residents at Georgetown University Hospital and adjusted based on feedback. The survey was then sent electronically to members of the AWS database. At the time of survey submission, the AWS database included 1,065 individual members composed of: 4 associate, 2 undefined, 59 emeritus, 53 new surgeon, 44 lifetime, 18 missionary, 488 regular, 182 student, and 215 resident members. Information and access to the database was obtained through communication with the AWS executive director. In order to understand current perceptions of those selecting a specialty and undergoing training for surgery, both current medical students and residents were considered ‘still in training’. Participants were asked to select ‘not applicable’ if a question pertained to experiences or observations beyond their level of training. The survey was distributed twice to the database, with the second distribution within 6 months of the first.

The study was approved by the Georgetown University Institutional Review Board. Information regarding the purpose of the study and use of responses was provided in an electronic cover letter sent to members of the database and again at the beginning of the survey link. We obtained informed consent from participants in the first question of the survey. Participants who chose not to consent were routed out of the online survey based on their answer.

### Survey design

We used a cross-sectional study design whereby members of AWS's database received a 19-page electronic survey to complete using SurveyMonkey^®^ (www.surveymonkey.com). Each respondent was assigned a unique identification number by the survey account. This unique number was linked with each response provided by the respondent.

Based on published recommendations, the survey included 42 fixed-response multiple choice, yes or no, and Likert scale questions with one additional open-field comment box (17). Not all 334 respondents answered all questions due to the use of question skip logic and respondent fatigue. After providing demographic data, respondents were asked to indicate on a Likert scale the degree of prevalence of discrimination in medical school, residency training, and practice. Gender-based discrimination was defined as harmful actions or utterances against a woman undertaken by either a man or a woman. Respondents were asked to indicate the sources of both experienced and observed discrimination, the recipient of the observed discrimination, frequency and type of discrimination, and setting in which discrimination was most prevalent. As experiences may have occurred more than once, multiple responses were allowed. We used a Likert scale to assess to what extent respondents believed various occupational factors were affected or unaffected by discrimination. Respondents were also asked questions regarding reporting of experienced and observed discrimination. All ‘not applicable’ responses were omitted from analysis. Finally, we included an open-field comment box to allow respondents to write free-text comments on their experiences with discrimination.

### Grounded theory

The 55 personal experiences provided in the survey comment box were analyzed using Strauss and Corbin's reformulated grounded theory. This theory entails first, coding and systematically analyzing data to verify a hypothesis and second, inspecting data for properties of categories from which theoretical ideas can be generated (18–20). Data is broken down, compared, and categorized using both inductive and reductive processing. We used an open, axial, and selective coding analysis to construct our theoretical framework. Based on this framework, personal anecdotes were categorized based on source and type of discrimination. Same-sex and inter-sex discrimination reports were included when subjects mentioned the actions of a particular sex in their response. Comments were classified specifically into categorical themes based on the analyzed content of the anecdote. Relative frequencies of each category and theme were calculated to better understand the prevalence of specific types of discrimination in each population subgroup.

### Data analysis

We calculated demographic frequencies (Table 1), and determined frequency of gender-based discrimination by stage of training (Table 2). Responses indicating some to very frequent (Likert scale 5–10) gender-based discrimination were considered significant whereas those indicating some (Likert scale 1–4) gender-based discrimination were considered insignificant. Reported sources and recipients of experienced and observed gender-based discrimination were tallied and stratified by gender (Table 3). Finally, types of gender-based discrimination were stratified by order of frequency (Table 4). All analyses were performed using SPSS Statistics (IBM Software).

**Table 1:** Demographic distribution of survey respondents^a^

	*N*	%		*N*	%
Age			Ethnicity		
< 25	1	<1	White	257	77
25–30	63	19	African American	9	3
31–35	84	25	Hispanic/Latino	8	2
36–40	38	11	Asian	37	11
41–45	32	10	Native American/Pacific Islander	1	<1
> 45	116	35	Combination	22	7
Marital status			Geographic region of practice		
Single	106	32	Northeast	98	29
Married	196	59	Southeast	60	18
Widowed	1	<1	Midwest	93	28
Divorced	23	7	Pacific Northwest	33	10
Separated	1	<1	Southwest	39	12
Civil Union	7	2	International	11	3
Surgical specialty			Years in practice		
General surgery	213	64	0 (please select if still in training)	126	38
Breast	46	14	1–5	52	16
Cardiothoracic	16	5	6–10	33	10
Transplant	12	4	11–15	39	12
Vascular	24	7	16–20	22	7
No response	23	7	21–25	28	8
Practice settings			26–30	16	5
Rural/small community hospital	25	7	> 30	18	5
Managed care/HMO	12	4	Still in training		
VA hospital	18	5	Yes	133	40
Academic/University hospital center	213	64	No	204	60
Private hospital	86	26			
No response	12	4			

a Subjects indicated demographic information; only female subjects were included in data analysis.

**Table 2:** Degree of frequency of experienced and observed discrimination^a^^,^ b

	Experienced discrimination		Observed discrimination	
		
	*N*	%	*N*	%
Significant frequency				
Medical school	172	53	157	54
Residency training	204	67	161	64
Practice	125	68	88	54
Insignificant frequency				
Medical school	107	33	76	26
Residency training	63	21	52	21
Practice	41	23	39	25
No frequency				
Medical school	44	13	53	19
Residency training	36	12	39	15
Practice	18	9	32	21

a Subjects indicated the frequency of experienced or observed discrimination during each training period; they were permitted to indicate only one frequency for each training period.

b Significant, insignificant, and no frequency categories were determined based on ratings indicated on a Likert scale.

**Table 3:** Perceived sources and recipients of discrimination^a^

Source of perceived discrimination, experience	Male		Female		Source of perceived discrimination, observation	Male		Female		Recipient of perceived discrimination, observation	Male		Female	
					
	*N*	%	*N*	%		*N*	%	*N*	%		*N*	%	*N*	%
Administrative staff	134	6	173	12	Administrative staff	103	6	131	12	Administrative staff	2	<1	83	5
Attending physician	508	23	117	8	Attending physician	426	25	91	9	Attending physician	17	7	230	13
Clerical staff	60	3	213	14	Clerical staff	45	3	147	14	Clerical staff	6	3	94	5
EMS staff	107	5	58	4	EMS staff	74	4	31	3	EMS staff	3	1	24	1
Intern	152	7	42	3	Intern	122	7	35	3	Intern	32	14	291	16
Medical school professor	191	9	28	2	Medical school professor	165	10	27	3	Medical school professor	8	3	74	4
Medical student	146	7	46	3	Medical student	109	6	33	3	Medical student	53	23	311	17
Nursing staff	94	4	428	29	Nursing staff	92	5	317	30	Nursing staff	29	13	178	10
Patient	460	21	268	18	Patient	295	17	171	16	Patient	29	13	101	6
Resident	314	16	98	7	Resident	274	16	81	8	Resident	51	22	398	22
Total instances of perceived discrimination	2166		1471		Total instances of perceived discrimination	1705		1064		Total instances of perceived discrimination	230		1784	
Percentage (%) of total instances of perceived discrimination (*N*=3637)	60		40		Percentage (%) of total instances of perceived discrimination (*N*=2717)	63		37		Percentage (%) of total instances of perceived discrimination (*N*=2014)	11		89	

a Subjects indicated whether or not they experienced or observed discrimination from a male, a female, or both; they were permitted to indicate multiple sources or no sources for each category.

**Table 4:** Order of indicated frequencies of experienced types of discrimination^a^

		No experience			Five or less total experiences			Annual experience			Monthly experience			Weekly experience	
										
		*N*	%		*N*	%		*N*	%		*N*	%		*N*	%
1.	Inappropriate firing from position	127	25	Barriers to promotion or barriers to job progression	52	15	Disparities in salaries or benefits	41	26	Inappropriate verbal exchange	25	29	Lack of respect from medical team	25	23
2.	Barriers to hire for applied positions	92	18	Lack of respect from medical team	50	15	Lack of respect from medical team	31	17	Lack of respect from medical team	16	18	Inappropriate verbal exchange	24	22
3.	Bias against pregnancy	75	15	Sexual harassment	49	15	Inappropriate verbal exchange	30	17	Disparities in salaries or benefits	13	15	Disparities in salaries or benefits	21	19
4.	Sexual harassment	66	13	Inappropriate verbal exchange	47	14	Barriers to promotion or barriers to job progression	30	17	Barriers to promotion or barriers to job progression	12	14	Barriers to promotion or barriers to job progression	12	11
5.	Disparities in salaries or benefits	48	9	Barriers to hire for applied positions	46	14	Sexual harassment	20	11	Sexual harassment	9	10	Bias against pregnancy	11	10
6.	Barriers to promotion or barriers to job progression	47	9	Bias against pregnancy	44	13	Bias against pregnancy	16	9	Bias against pregnancy	7	8	Sexual harassment	9	8
7.	Lack of respect from medical team	31	6	Disparities in salaries or benefits	30	9	Barriers to hire for applied positions	7	4	Barriers to hire for applied positions	4	5	Barriers to hire for applied positions	4	4
8.	Inappropriate verbal exchange	27	5	Inappropriate firing from position	18	5	Inappropriate firing from position	4	2	Inappropriate firing from position	1	1	Inappropriate firing from position	3	3

a Subjects indicated the frequency of each experience type based on the above five categories; subjects were permitted to indicate more than one type of experience for a given temporal category.

## Results

Our overall survey response rate was 31% (343 out of 1,105). Of the 343 total survey respondents, 334 identified themselves as women and were used for data analysis. Of the respondents, 40% are still training, 77% are white, 59% are married, and 65% are under the age of 45. Geographic location, surgical specialty, and practice setting are reported in Table 1. The majority of respondents practice or intend to practice general surgery in an academic hospital/university medical center setting.

Fifty-three percent, 67%, and 68% of respondents experienced some to very frequent discrimination, whereas 54, 64, and 54% reported observing some to very frequent discrimination during medical school, residency, and practice, respectively (Table 2). Only 19, 15, and 21% reported no observation of gender-based discrimination in medical school, residency training, and practice, and of note, only nine respondents who are still in practice indicated that they had never experienced gender-based discrimination. Reported degrees of discrimination by those still in training parallel those of the greater study population.

Those who experienced discrimination reported 60% of sources as male and 40% as female (Table 3). Similarly, 63% of observed sources of discrimination were male and 37% were female. The majority of observed recipients of gender-based discrimination were female (89%), however, some (11%) were male. Sources of gender-based discrimination are delineated in Table 3. Interestingly, although most sources of gender-based discrimination reflected 60% male versus 40% female, administrative staff, clerical staff, and nursing staff sources of gender-based discrimination were more likely to be female.

Respondents were asked to report frequency for each of the seven types of discrimination listed in Table 4. One hundred eighty-one respondents did not answer this question. The most frequent reports were inappropriate verbal exchanges, disparities in salaries or benefits, and a lack of respect from the medical team, based on ranked order of reported frequency, from weekly to never experiences (Table 4). Types of discrimination with the highest indicated frequencies were similar for both experienced and witnessed events. Inappropriate firing from a position and barriers to hire for applied positions were rarely reported.

Respondents were also asked to report work settings in which gender-based discrimination was experienced or observed (data not shown). Sixty-seven percent reported that discrimination occurred with colleagues/referrals, 66% in inpatient care settings, and 62% in the operating room. Seventy-seven percent of observed discrimination occurred in inpatient settings, 75% in the operating room, and 71% with colleagues/referrals.

Sixteen percent of respondents reported personal experiences in the open-ended comments section. There were 36 specific references of inter-sex discrimination, 17 of same-sex, 28 did not reference gender, and 5 referenced both. There were 46 negative overt reports, such as sexual harassment, and 41 negative covert reports, such as expectations of higher performance (double standard). Ten comments did not provide specific details of discrimination. There were three positive reports of women surgeons receiving supportive treatment. Negative themes of both same- and inter-sex gender discrimination included derogatory comments and discouragement, 9; intimidation or silencing tactics, 9; double standard and/or higher expected standards for women surgeons, 8; unequal pay and/or benefits or career advancements, 5; persistence of the ‘old boys’ club’, 4; problems with maternity-leave policies, 4; overt sexual harassment, 3; and discrimination from patients, 2. Importantly, nine respondents described avoidance behavior, including leaving the medical profession, to evade continued experience with discrimination.

Among those who experienced gender-based discrimination, 30% indicated they had reported discrimination to a coworker, supervisor, or ombudsperson. Twenty-seven percent of those who witnessed gender-based discrimination reported the incident. Out of the respondents who had experienced or observed gender-based discrimination and reported the incident, 35 and 37%, respectively, indicated that action had been taken to address the incident. Additionally, 16 and 7% of respondents who indicated that they had experienced or observed gender-based discrimination, respectively, requested a position or unit reassignment during training or practice and 45% considered leaving or declining a position either during their medical training or career. Twenty-four percent and 14% of respondents who experienced or observed discrimination, respectively, did leave or decline a position because of this discrimination. Respondents reported that job satisfaction, coworker/colleague respect, and career advancement were the areas most affected by gender-based discrimination.

## Conclusions

With women representing a smaller number of both practicing and training surgeons, it is important to understand why women are less likely than men to apply to careers in surgery and its subspecialties. Previous claims have suggested that lifestyle and family preferences are the primary deterrents for women entering surgery, yet women are actually less likely than men to cite family concerns or workload as deterrents. Perception of gender-based discrimination and of the traditional ‘old boys’ club’ have been implicated as more prevalent deterrents (4–6). Our study suggests that gender-based discrimination – stemming from both women and men – persists and has broad implications for women choosing a career in surgery.

Despite the strides that have been made in gender equality over the past century, more than half of our respondents reported experienced or observed gender-based discrimination. Gender-based discrimination appears to persist in all forms of training and practice, is diverse, and exists across a spectrum of practice settings and specialties. Female medical students have a more negative understanding of the professional and personal life conflict present in a career in surgery in comparison to female surgeons who reported positive feelings about their career in surgery (2). This discordance represents a misunderstanding in female medical students regarding the realities of a life in surgery and appears to be strengthened by the notion that male attendings have a less favorable perception that surgery is a good career for women (3). As such, it seems that institutions may lack the foundation to support women in surgery. Only 9% of female surgeons still in practice have never experienced gender-based discrimination, whereas almost half of those who have experienced or observed gender-based discrimination have considered leaving or declining a position during training or practice as a result. Measures to address and limit gender-based discrimination in the surgical workplace are clearly needed.

Our study findings shed new light on this complex and emotionally charged issue, demonstrating the need for education and change. First, although the majority of sources of perceived discrimination were reported as men (male attending physicians being highest), 40% of both experienced and observed gender-based discrimination came from women. In fact, among administrative, clerical, and nursing staff, women were more likely to be reported as sources of gender-based discrimination. The rationale for this same-sex bias is unclear, yet is not specific to medicine. Women find women in leadership positions to be less qualified and less desirable than identically described men (21–25). In cross-occupational studies, successful women in male domains were less liked and more likely to be attributed to undesirable interpersonal qualities (23–25). Interestingly, these undesirable qualities, often assertive and agentic, are considered favorable within the context of male leaders, whereas communal and nurturing attitudes are more successful for females (25). Females enter the workplace with more hesitancy to exert authority yet expect other female colleagues to meet higher expectations, but this unconscious discordance in expectations causes resentment (25, 26). Within the male-dominated world of surgery, where women are represented by low numbers, women discriminating against women may perpetuate the cycle of gender disparity. It has been proposed that there is a dynamic between the female nurses’ nurturing characteristics, which are traditionally feminine, and the female surgeons’ fight to attain agency as a leader, often requiring calculated cooperation in the workplace (25, 26). Educating women, particularly administrative, clerical, and nursing staff working with women surgeons, could dramatically and positively impact discrimination in the surgical workplace.

Second, we found that significant gender-based discrimination occurred throughout training (medical school and residency) into surgical practice, and involved a broader spectrum of locations and sources than previously identified (5, 6, 27). To be most effective, programs to raise awareness of gender-based discrimination and promote gender equality in the surgical workplace should occur throughout training and as part of continuing medical education and should reach operating room, inpatient, administrative, clerical, and nursing staff as well as physicians, teachers, and students. Although some argue that students vary in their perception of mistreatment and discrimination, formal education addressing issues of mistreatment and sensitization for both teachers and students can improve team communication and optimize the surgical environment (13). The open-ended responses that we received suggested specific areas to be rectified. Although some disparities in salaries and benefits or unwieldy maternity-leave policies are more easily addressed, other common themes, like inappropriate verbal exchanges, lack of respect from the medical team, biased referral patterns, discouragement, and intimidation, are more covert and may be more difficult to remediate.

Third, although survey respondents stated that gender-based discrimination negatively impacts job satisfaction, perceptions of self-efficacy, coworker/colleague respect, and career advancement, a minority reported it to colleagues or supervisors. Other studies have shown that many residents find reporting abuse to be more troublesome to their careers than non-reporting, worsening job satisfaction (9). Of those survey respondents who reported, the majority described a lack of action as the result. Medical students throughout their training report they feel the need to adapt their identity to fit a more androgynous image by adopting a ‘no-emotion, no-fear’ response within their male-dominated teams (28). This is, in fact, a learned behavior from their female attendings and senior residents; by graduation, the majority of medical students have progressive desensitization to discrimination, learning to systematically tolerate discrimination as a part of their future career (28). This is congruent with other research findings on the reporting of gender-based discrimination and may be a reflection of the subtlety of gender-based discrimination in today's surgical workplace (5). Often, situations of sexual harassment are not identified by the recipients as such, which may have prevented proper reporting of such incidents; gradual acculturation and minimization of behaviors that are advocated by female superiors may contribute to the pattern of non-reporting currently seen in medicine (28). As such, the current landscape proposes that women entering the surgical field may have to simply deal with this behavior if they wish to assimilate.

Fourth, although respondents from non-academic settings also indicated experience with or observation of discrimination, the majority of respondents trained, worked, or intended to practice in an academic setting. These findings support prior claims that although discrimination is not specific to academic medicine it does persist in university settings (5–7, 29). As such, academic centers are uniquely positioned to serve as standard bearers for this pervasive issue. Effective university-based gender-based discrimination programs could be scaled to smaller community settings.

Our study, although revealing novel and important insights into the breadth, timing, settings, sources, and gender of perceived discriminatory influences, has some limitations. Although our response rate is consistent with other electronic physician surveys, it is low (28). Both study length and the fact that it was sent electronically and only twice likely contributed to our low response rate. Validated surveys were unavailable for use. As the AWS is a professional organization that focuses on advancing the goals of women in surgery and neutralizing gender bias, use of its members as participants adds selection bias to the study. The majority of respondents practiced in an academic university hospital or medical center, reflective of the AWS membership, which limits the generalizability of our results. Only nine respondents had no experience with discrimination in training or practice, suggesting that only those adept at perceiving gender-based discrimination responded to the survey. Recall bias is also a relevant limitation, particularly considering the range of practice years of the participants. Last, though we identified women who perceived discrimination, we were unable to determine whether these perceptions were accurate. Although the authors recognize the sampling and recall bias involved in the study, the findings of the study are nonetheless valuable.

When women students hear about challenges for women in surgery or observe gender-based discrimination, they do not as easily imagine their future as successful (30). Mentorship and early exposure play a positive role in women's decisions to enter surgery, and a greater proportion of successful women in surgical departments should allow more female students to recognize surgery as a viable option (31). Of note, three women in our survey reported being positively supported based on their gender. Additionally, exploring how institutions can best help male physicians and trainees to mentor female trainees is worthwhile in order to truly equalize the surgical environment. Given that mentorships form when the junior and senior have common experiences, male physicians may have less success with female trainees as they are less open to work through female issues and often exhibit paternalism, where males withdraw as mentors when they feel the female mentee exhibits independence or appears self-sufficient (32). However, male mentees are more likely than female mentees to attract career sponsors, have more extensive and influential networks, and are more often perceived as competent and hirable based on acceptable agency (32). Both male and female mentors and faculty members need to recognize female mentees tendency to underestimate themselves and hesitancy to assert agency over communality within the context of stereotype threat in order to promote gender identity safety (32, 33).

Female medical students may avoid surgery because of perceived anticipated barriers and discordance between the perception and reality of career satisfaction. Also, students are often less able to negotiate their feelings and concerns within attendings and male residents if they feel uncomfortable (32). Institutions should consider formal education to prepare trainees to deal with the realities of surgery and to promote a sense of control over perceived inequality. Fostering arenas where both trainees and faculty members can both informally and formally discuss discrimination incidents or career concerns could help to both improve female's feelings of inclusion in the field of surgery and minimize feelings that discrimination is disregarded. Female surgeons report that, in terms of personal success, empowered mentorship remains the most important factor, followed by defining career goals and refining personal writing and speaking skills (16). Additional advice includes depersonalizing self from the impact of discrimination while focusing on perseverance, work ethic, courage, and a sense of humor in order to overcome obstacles to leadership within the surgical field (16). Providing mentorship from females within and outside of medicine as well as leadership training could improve female surgical trainees’ success in the field. Women with high leadership efficacy have decreased susceptibility to negative effects of stereotypes, such as gender discrimination (33).

Future directions should include investigating ways in which medical institutions are creating supportive programs for their women surgeons by creating systemic change to address negative patterns. Social support and social networks are successful implementations that have worked for both female surgeons as well as minorities within business and science and engineering (3). Scarcity of mentors can be improved institutionally by establishing these networks within the current female faculty and the regional and national groups, as well as recruiting mentors from non-medical departments to educate faculty on career advancement tactics (34). By considering the constraints of gender roles, such as family life, within the context of a surgeon's lifestyle, institutions can better accommodate the dual lives of their faculty (34). Additional studies might examine factors of resiliency that contribute to the success of women who remain in the field, the rationale for discrimination between women specifically in medicine, and the long-term implications of decreased job satisfaction at all levels of surgical training and practice.

Gender-based discrimination remains in flux. Although arguably less or more covert than in the past, it remains pervasive and not yet openly discussed or reported. Although discrimination may have been a societal norm for prior generations of women, today's generation of women choosing surgery does not expect discrimination. It is our hope that targeted interventions informed by our study outcomes will promote equality in the surgical workplace, throughout training and careers, and across a variety of surgical specialties and settings.
